# From consultation to choice: gynecologic cancer patients’ perspectives on shared decision making in clinical practice – findings from a cross-sectional observational qualitative interview study

**DOI:** 10.1186/s12910-026-01419-1

**Published:** 2026-02-24

**Authors:** S. Theis, H. Frühwein, A. Hasenburg, M. Moehler, N. W. Paul

**Affiliations:** 1 Department of Obstetrics and Gynegology, University Medical Center of Johannes Gutenberg University Mainz, Langenbeckstraße 1, Mainz, 55131 Germany; 2 Institute of History, Philosophy, and Ethics of Medicine, Johannes Gutenberg University Medical Center Mainz, Am Pulverturm 13, Mainz, 55131 Germany; 3 Department of Internal Medicine I, University Medical Center of Johannes Gutenberg University Mainz, Langenbeckstraße 1, Mainz, 55131 Germany

**Keywords:** Shared decision making, Gynecologic cancer, Participation, Qualitative research, Patient autonomy

## Abstract

**Background:**

Shared decision making (SDM) is a cornerstone of patient-centered care in oncology, yet its implementation remains inconsistent across clinical contexts. Particularly in gynecologic oncology, where treatment choices often carry profound implications for identity, fertility and quality of life, understanding how patients engage in SDM is crucial. Patient autonomy, understood as the capacity and right to participate meaningfully in medical decisions, is central to this process, yet remains complex and context-dependent.

**Objective:**

This study explores how women with gynecologic cancers experience decision making processes throughout the course of their treatment, with a focus on how their preferences, needs, and autonomy evolve over time.

**Methods:**

We conducted a qualitative, single-center, cross-sectional observational study using grounded theory methodology. Semi-structured interviews were held once with 20 female patients undergoing treatment for various gynecologic malignancies at a university medical center. Interviews explored experiences across treatment phases initiation, maintenance, pending/abstention and anticipated decisions.

**Results:**

Participants expressed a wide range of attitudes toward decision making, shaped by emotional readiness, trust in clinicians, and interpersonal support. Early in treatment, many preferred clinician-led guidance due to emotional overload. Over time, some sought greater involvement, while others continued to delegate decisions. A minority of participants addressed treatment refusal or end-of-life decisions during interviews, often with ambivalence. Structural issues such as fragmented communication and lack of care continuity were cited as barriers to meaningful participation.

**Conclusion:**

Patients’ preferences for involvement in decision making are dynamic and context dependent. Our findings support models of relational and temporal autonomy and highlight the importance of responsive communication, trust, and systemic support in fostering ethical and effective SDM.These insights call for enhanced clinician training and organizational strategies to integrate SDM meaningfully into routine cancer care.

**Supplementary Information:**

The online version contains supplementary material available at 10.1186/s12910-026-01419-1.

## Background

In 2006, the World Health Organization (WHO) emphasized the importance of empowerment as a strategy to improve health outcomes and quality of life, highlighting the need for positive cooperative relationships and self-care practices among patients [1]. Empowerment serves as a motivational framework that encourages individuals to take greater responsibility for their health decisions [2]. Rather than focusing on patient deficits, empowerment strategies aim to build on patients’ capabilities. In this context, healthcare professionals are seen not as authoritative decision makers but as collaborators who support the development of patients’ knowledge and self-management skills [3].

At the core of patient empowerment lies the ethical principle of autonomy- the right of individuals to make informed, voluntary decisions about their own care. In medical ethics, autonomy has traditionally been viewed as self-determination and independence. However, contemporary scholarship challenges this purely individualistic interpretation. Relational autonomy emphasizes that decision making occurs within a network of relationships - familial, social, and institutional - and that emotional readiness, trust, and access to resources are key enablers of autonomy [4, 5]. Temporal autonomy adds that patients’ preferences and capacities evolve over time, especially in chronic or high-stakes conditions such as cancer [5]. In this light, shared decision making (SDM) can be seen as a practical expression of both empowerment and autonomy. Defined as a collaborative process in which clinicians and patients jointly consider treatment options and make informed, value-based decisions [6], SDM is especially critical in areas like oncology, where interventions often involve significant trade-offs and affect deeply personal domains such as fertility, body image, life expectancy, and the ability to participate in divers social settings. By fostering patient - centred communication and mutual respect, SDM has been associated with improved patient satisfaction, enhanced comprehension of treatment options and alignment of clinical decisions with individual patient values and preferences [7–10].

Despite these benefits, evidence from clinical practice suggests that SDM is inconsistently implemented in routine care [11–13]. For example, a recent cross-sectional study in Japan evaluated SDM among patients with gynecologic cancer and healthcare professionals [14]. The study revealed that awareness of SDM practices varied significantly among healthcare providers: 24% of physicians, 48% of nurses, and only 20% of pharmacists were familiar with SDM. Additionally, a misalignment in treatment goals between healthcare providers and patients was observed. Namely patients prioritized complete cancer elimination, while healthcare professionals more often emphasized life prolongation. Although widely recognized as essential to patient-centered care, SDM is therefore often impeded by practical barriers, leaving a persistent gap between patient preferences, achievable goals, and real-world clinical decisions.

In a Danish study the implementation of two patient decision aids (PtDAs) for women with recurrent ovarian cancer across three oncology departments was evaluated [15]. The study found a significant improvement in observed SDM practices post-implementation (*p* = 0.002). Notably, enhancements in SDM were evident in consultations where physicians had received more than two hours of SDM training (*p* < 0.001), whereas no significant changes were observed with less training. The intervention improved observer-rated SDM in consultations, whereas patient-reported and physician-reported SDM as well as the physicians’ treatment recommendations did not change [15].

Glyn Elwyn and colleagues proposed a structured, three-step model to embed SDM into routine clinical care in 2012, offering a conceptual framework to guide collaborative healthcare decision-making [6]. In 2017 the model was revised by the same authors as the ‘three-talk model’ to suit everyday clinical practice even better [16]. The model consists of “team talk” (invitation of the clinician to the patient into a partnership to find best suitable decision), “option talk” (describing available options and alternatives and their risks/benefits), and “decision talk” (supporting the patient in weighing preferences to reach a decision). Further developments can be expected in the near future based on an abstract that has already been published: this describes the process of SDM as reflective and deliberative rather than prescriptive, with potential benefits for both patient care and clinician well-being [17]. Rooted in communication theory, the model positions clinicians as facilitators of informed, values-based choices, rather than sole decision makers. The authors argue that SDM improves patient satisfaction, treatment adherence, and ethical standards, though they acknowledge barriers such as time constraints and the need for training. They advocate for broader adoption through integration into clinical practice guidelines and policy frameworks. Further insights come from the recent Health Technology Assessment (HTA) report 2024 by the German Institute for Quality and Efficiency in Health Care [18]. The report evaluated the effectiveness of SDM interventions. In contexts where multiple medically reasonable options exist, SDM is most valuable, meaning that patient choices (guided by their values) do not necessarily alter clinical outcomes. Its true benefit lies in enhancing patient- centred outcomes: knowledge, involvement, reduced decisional conflict and decisions aligned with personal preferences. Interpreting limited effects on morbidity or mortality as failure of SDM, as done in the report, risks misunderstanding the purpose of SDM. These findings strongly support the necessary systematic integration of SDM into routine clinical practice as a standard of high-quality, patient-centered care.

Although SDM has been studied predominantly in breast cancer with multiple interview-based qualitative studies [19–23] - documenting patients’ diverse decision styles, information needs and responses to decision aids - qualitative research specifically addressing SDM across gynecologic cancer types is still limited and often topic-specific, for example, fertility or health-literacy focused [24, 25]. SDM remains under-investigated from the patient perspective [22, 23, 26], while provider interviews highlight organizational constraints on SDM implementation.

Although SDM is considered as a benchmark of high-quality in patient-centered oncology care, little research has examined how women with gynecologic cancers experience decision making across the treatment trajectory. Addressing this gap, the present study investigates from patients’ perspective how women diagnosed with gynecologic cancers (histologically confirmed ovarian, breast, cervical or uterine cancer) experience decision making at different stages of disease: initiation phase, maintenance phase, pending or abstention. Our aim was to examine whether, and to what extent, patients’ lived experiences of decision making in oncology care corresponded with the normative and theoretical ideals of shared decision making, given that such ideals are not yet routinely realized in clinical practice.

## Methods

### Study design

This study is a cross-sectional observational sub-study embedded within a broader research project funded by the German Cancer Aid Foundation (Deutsche Krebshilfe). The overall project aims to identify and synthesize patients’ personal values, therapeutic goals, and their expectations - both positive and negative - regarding social participation, identity, and self-concept during cancer treatment using semi-structured qualitative interviews in female patients with gynecological tumors and male patients with gastrointestinal tumors.The present sub-study focusses on the female sub-cohort with gynecological tumors and investigates how these patients engage in SDM at three key decision points: initiation or escalation of current therapy (inititation / pending phase), continuation or adjustment of treatment (maintenance phase), and decisions to abstain from or discontinue treatment (abstention phase).

Given the complex and highly individualized nature of these experiences, a qualitative study design was chosen. Such experiences are difficult to capture with quantitative measures whereas qualitative methods allow for an in-depth exploration of subjective meanings shaped by individual contexts and enable the identification of emergent themes. The “Consolidated criteria for reporting qualitative research (COREQ)” were used as reference for the study (Supplementary file 2) [11]. 

### Setting and participants

Participants for this sub-study were recruited from affiliated gynecologic oncology department at a University Medical Center in Germany at the point of therapy initiation, transition into or during maintenance therapy, following the finalization of individualized treatment plans by the respective interdisciplinary tumor boards once the therapeutic regimen had been formally confirmed and prior to the baseline research visit. Participants were eligible for inclusion (see Table 1) if they were adult women (≥ 18 years) with a histologically confirmed initial diagnosis of gynecologic cancers (including ovarian, breast, cervical or uterine). All participants were required to have full decisional capacity and the ability to provide informed consent. Patients receiving acute psychiatric treatment for moderate to severe conditions that may interfere with study participation or affect primary psychosocial outcomes or with cognitive impairments that could interfere with consent or meaningful participation were excluded to ensure valid informed consent. Likewise, patients of advanced age whose treatment was guided primarily by geriatric oncology assessments and age-specific therapeutic goals were not eligible. This criterion must be understood in the context of the overarching study concept [27]. In line with this overarching concept, which aimed to compare younger female with older male oncologic patients, an upper age limit of 75 years was defined for the gynecological cohort to ensure comparability between groups, consistency in the clinical decision making frameworks and a methodologically homogenous study population.

**Table 1:** Eligibility criteria

Gynaecological tumors	
Inclusion criteria	Exclusion criteria
Female	Patients receiving acute psychiatric treatment for moderate to severe conditions that may interfere with study participation or affect primary psychosocial outcomes
Age ≥ 18	Advanced age who are treated on the basis of gerontological oncology assessments and age-appropriate treatment goals
Histologically confirmed gynecological cancer (ovarian, breast, cervical, uterine)	Cognitive impairment
Capable to give consent	

### Materials

Data was collected through a short demographic questionnaire (age, gender, marital status, children, religion, highest level of education) and semi-structured, in-depth interviews guided by a thematically structured interview framework developed specifically for this study (see supplementary file 1) [27]. The guide encompassed three major domains: (1) bodily and physical experiences of illness, (2) individual therapy goals and treatment-related decision making, and (3) expectations - both positive and negative - regarding social participation, identity, and self-concept. The interview structure was designed to facilitate narrative exploration, with open-ended questions and flexible prompts tailored to the participant’s clinical and emotional context. The questionnaire did not contain any explicit questions about decision making.

### Ethics approval and pre-registration

The study was prospectively registered with both the Research Registry (Registeration ID: 6615128527f813002936e32b) and the German Clinical Trials Register (DRKS00033242). Ethics approval was obtained from the Ethics Committee of the State Medical Association of Rhineland-Palatinate, Germany (approval no. 2022–16670, version 4.0, dated 23 November 2022, decision issued on 14 September 2022). Approval was obtained through this committee, which is the responsible ethics committee for physician-led research affiliated with institutions in Rhineland-Palatinate, including University Medical Center Mainz (Johannes Gutenberg University Mainz). The trial was conducted in full accordance with the Declaration of Helsinki and relevant national data protection regulations (DSGVO).

### Data collection

Suitable participants were approached on site or contacted by telephone by the lead interviewer. Three suitable participants refused study enrollment (no time/ too many appointments/ not interested). After receiving detailed information about the study’s objectives, procedures, and their rights as research participants, all participants provided written informed consent. They were explicitly informed of their right to withdraw from the study at any time without providing a reason and without any consequences. Interview appointments were scheduled to suit the patients: some preferred to perform interviews during their therapy days while others preferred to have an extra appointment.

A total of 20 participants were enrolled between December 2023 and April 2025. All participants had received a histologically confirmed diagnosis of a gynecologic malignancy (ovarian, cervical, uterine, or breast cancer) and were either preparing to initiate therapy (initiation phase), awaiting the start of therapy (pending phase), actively abstaining from therapy (abstention phase), changing therapy regimen, or undergoing maintenance therapy (maintenance phase). Each participant was interviewed once, using the mentioned semi-structured interview guide. The interview structure was particularly designed to facilitate narrative exploration, with open-ended questions and flexible prompts tailored to the participant’s clinical and emotional context.

Interviews were conducted in person by the lead interviewer, a clinician-researcher and board-certified gynecologist. Throughout the interview process, reflexivity was maintained to balance professional familiarity with methodological openness and neutrality. Each interview was audio-recorded using a digital recording device with written informed consent obtained in advance. The recordings were transcribed verbatim and pseudonymized during the transcription process to ensure participant confidentiality. In addition to the recordings, field notes were documented after each interview to capture non-verbal cues, emotional responses, and contextual details that might inform later stages of analysis.

### Data analysis

Transcripts were analyzed using principles of grounded theory methodology, following the iterative and comparative logic outlined by Charmaz et al. 2017 [28, 29]. This approach enabled the development of categories and conceptual insights directly rooted in participants’ narratives, with a particular focus on their perceptions of shared decision making, personal treatment goals, and psychosocial impacts of cancer and therapy.

Transcripts were uploaded and coded using MAXQDA Analytics Pro (VERBI Software, 2022), a qualitative data analysis software that facilitated systematic coding, memo writing, and comparative data organization. The analysis proceeded in three stages (see Table 2 for the coding tree):

**Table 2:** Coding tree with frequencies (segment counts) and SDM-phase classification

Level 1 (Domain)	Domain total (segments)	Level 2 (Open code)	Code frequency (segments)	Level 3 (SDM phase)
Physical impact	161			
		Illness-related bodily impact	32	Initiation
		Sense of having cancer (bodily perception)	14	Initiation
		Side effects of therapy	63	Maintenance / Adaptation of therapy
		Coping with therapy side effects	52	Maintenance / Adaptation of therapy
Individual limitations (reflexive)	35			
		Limitations due to illness	6	Initiation
		Limitations due to therapy / side effects	18	Maintenance / Adaptation of therapy
		Long-term consequences of therapy	11	Pending
Treatment setting	179			
		Specific structural conditions	16	Initiation
		Negative experiences with procedures	53	Maintenance / Adaptation of therapy
		Positive experiences with procedures	43	Maintenance / Adaptation of therapy
		Negative experiences with staff	25	Maintenance / Adaptation of therapy
		Positive experiences with staff	42	Initiation
Coping strategies	148			
		Rational coping	81	Maintenance / Adaptation of therapy
		Spiritual coping	8	Pending
		Psychological coping	47	Maintenance / Adaptation of therapy
		Physical coping	12	Maintenance / Adaptation of therapy
Goals	30			
		Primary goals	17	Initiation
		Secondary goals	13	Maintenance / Adaptation of therapy
Emotional reactions	177			
		Hope	18	Pending
		Gratitude	16	Maintenance / Adaptation of therapy
		Trust	9	Initiation
		Shame	1	Maintenance / Adaptation of therapy
		Guilt	5	Initiation
		Sadness	12	Abstention / End of treatment
		Anger	6	Maintenance / Adaptation of therapy
		Fear	59	Initiation
		Disappointment	26	Maintenance / Adaptation of therapy
		Concern about reduced social participation	11	Maintenance / Adaptation of therapy
		Feeling in social situations	14	Maintenance / Adaptation of therapy
Social participation (relational)	144			
		Social resources for coping	50	Maintenance / Adaptation of therapy
		Reduction of social participation	10	Maintenance / Adaptation of therapy
		Restriction of social participation due to illness	15	Maintenance / Adaptation of therapy
		Reaction of the environment	69	Initiation
Body image / self-perception	65			
		Influence of illness on sexuality	9	Maintenance / Adaptation of therapy
		Change of body image as a woman due to illness	19	Maintenance / Adaptation of therapy
		Change of body image due to illness	17	Maintenance / Adaptation of therapy
		Self-image	20	Maintenance / Adaptation of therapy
Other	10

1. Initial (open) coding: Transcripts were read line-by-line and coded inductively to capture participants’ language, metaphors, and expressed meanings by three assigned members of the research team. Codes remained close to the data and included, for example, expressions of decision pressure, trust and delegation, uncertainty and fear of consequences, information overload, active information seeking, and perceived roles in the consultation.
2. Focused coding: The most significant and frequently occurring initial codes were refined, merged, and differentiated into more conceptual focused codes through constant comparison within and across interviews. This step aimed to stabilize meanings, clarify code boundaries, and reduce redundancy while preserving nuance in participants’ accounts.
3. Theoretical (integrative) coding: Relationships between focused codes and categories were examined to construct higher-order themes and integrative conceptual insights. Particular attention was paid to how decision making was described as unfolding over time and across clinical contexts.

Throughout the analytic process, constant comparison was employed - codes and themes were compared across cases to identify variations, patterns, and underlying structures. Memos were used to document analytic decisions, track evolving interpretations, and reflect on the research team’s positionality Coding and theme development were conducted collaboratively by multiple members of the research team in interpretation meetings to enhance reflexivity and analytic rigor. Any discrepancies in coding were discussed and resolved through iterative consensus building.

For the purposes of the present manuscript, we conducted an SDM-focused analytic strand. After focused coding, decision-making and SDM-related codes were identified and organized into four therapy-phase groupings reflecting when and how decision situations occurred across the treatment trajectory: (1) initiation of therapy, (2) maintenance/adaptation of therapy, (3) pending decisions, and (4) abstention/end of treatment. These phase groupings (see Table 3.) represent higher-order analytic categories that integrate multiple SDM-relevant focused codes (rather than a complete representation of the full code system developed across all interview domains). The Results section provides illustrative quotations to show how participants’ accounts were coded and how these codes informed the phase-based categorization.

**Table 3:** Decision/SDM strand summary (Focused/theoretical coding output)

Final SDM/Decision category (therapy phase)	Frequency (*n*)	What is included (brief description)	Examples of contributing open-code domains (from Table 2)
Initiation	18	Decision situations at therapy start (e.g., initiating systemic therapy; weighing benefits/risks; role negotiation; information needs).	Emotional reactions; Treatment setting; Physical impact; Goals
Maintenance / Adaptation of therapy	20	Decision situations during ongoing therapy (e.g., managing side effects, dose changes, switching regimens; reassessing goals).	Physical impact; Coping strategies; Treatment setting; Goals
Pending	7	Decision situations characterized by postponement/deferral (e.g., waiting for test results; uncertainty; temporary non-decision).	Emotional reactions; Treatment setting
Abstention / End of treatment	3	Decision situations involving stopping/withholding therapy (e.g., treatment discontinuation; end-of-treatment considerations).	Goals; Physical impact; Emotional reactions

*This table summarizes the final SDM-related therapy-phase grouping (focused/theoretical coding output) derived from the broader initial/open coding system shown above*

Data collection continued until theoretical saturation was reached - that is, when additional interviews no longer yielded new conceptual categories or altered the understanding of emerging themes [30]. The grounded theory approach allowed the resulting categories to remain closely aligned with patients’ perspectives while also generating theoretical contributions concerning the practice and perception of shared decision making in gynecologic oncology.

Summarized, we conducted a cross-sectional observational study using semi-structured qualitative interviews employing a mixed-methods approach, integrating qualitative and quantitative research methodologies. This approach enabled a contextualized, process-oriented analysis of patients’ decision making experiences over the course of their cancer journey. Grounded theory methodology guided the qualitative component, allowing for inductive theory development based on patients’ lived experiences.

### Researcher characteristics

The research team represented a range of professional backgrounds leading to broad – based knowledge that informed the design, conduct, and interpretation of the study. ST, a gynecologist with additional designation in drug-based tumor therapy was trained in qualitative research methods, performed recruitment of the participants, conducted the interviews and was centrally involved in coding and data analysis. HF, experienced researcher in social sciences and qualitative research, contributed to coding and data analysis. AH, focus in area of gynecological oncology and co-principal investigator, was engaged in the conception of the study and provided critical feedback throughout the course of the study. MM, specialist for gastrointestinal tumors served as co-principal investigator, contributed to the study conception and provided critical feedback throughout the course of the study. NWP, principal investigator with expertise in ethics, oversaw the overall study design, guided the analytic process and provided critical feedback throughout the course of the study.

Reflexivity was an integral part of the research process. ST, as interviewer and primary analyst, reflected on how her professional role as a gynecologist could shape both interactions with participants and interpretation of data. HF and NWP brought ethical perspectives that encouraged systematic consideration of values and potential biases in data analysis. The involvement of AH and MM, who contributed clinical perspectives without participating directly in data collection, provided additional distance and opportunities for critical review. Regular team discussions across these different disciplinary backgrounds fostered reflexivity, challenged assumptions, and strengthened the credibility and trustworthiness of the findings.

## Results

### Sample characteristics

A total of twenty female participants, aged between 31 and 72 years (median 50.5 years), were included in the gynecologic sub - cohort of the study. The majority were married (65%), while 10% were divorced and 25% single. 60% of the participants reported having children (range: 1 to 4). In terms of educational attainment, 30% had completed university levels, 20% had a high school diploma (13 years of school), 40% had an intermediate school-leaving certificate (ten years of school), while 5% had a primary school or technical college qualification (four to nine years of school). Ten participants were in initiation phase, three in maintenance phase, four in pending (initiation to maintenance) and three in escalation phase of their treatment.

For the present analysis 48 codes within the data were identified that directly related to decision making and allocated to four catgeories: initiation of therapy (*n* = 18), maintenance/adaptation of therapy (*n* = 20), abstention/refusal or end-of-treatment choices (*n* = 3) and pending decisions (*n* = 7). Decision making codes were present in 70% (*n* = 14) of the interview transcripts, while 30% (*n* = 6) did not include any such references (see Table 2). Among the subcategories, 55% addressed decisions made at the beginning of the therapy (initiation), 45% related to ongoing therapeutic adaptation (maintenace), 15% pertained to refusal or cessation of treatment (abstention), and 25% captured prospective or anticipated decisions (pending).

### Initiation phase

During the initial phase of diagnosis and treatment planning, eleven participants talked about decision making experiences. A predominant theme among their narratives was a strong appreciation for clear, directive guidance from healthcare providers. Many participants expressed relief and gratitude for receiving structured and concrete recommendations regarding diagnostic and therapeutic steps. This reflects a tendency to favor clinician-led approaches during emotionally overwhelming moments, where the burden of autonomous decision making might feel excessive.

Several statements (*n* = 7) emphasized agreement with a more predetermined treatment path. For example, one participant explained that she was comfortable following the proposed therapeutic sequence without desiring additional decision making freedom. Another described feeling reassured by their gynecologist’s clear communication and felt adequately informed without needing further detail, saying, “*I have to say that I was very happy with my gynecologist. Because she really supported me well. She really told me what could happen. She knew herself that I was going to have chemotherapy. But she really told me what could happen. And I thought that was really good”* (Gyn_8).

In some cases, it was mentioned that women felt overwhelmed by too much freedom in choosing treatment options. They acknowledged that guidance provided a sense of security, especially in the face of uncertainty or limited understanding of medical complexities. Some also noted that, even with more information, they were unlikely to have made different choices - indicating a preference for trust-based delegation rather than self-directed decision making.

In response to the interwier’s question: *“Would you have liked more information? Or more freedom of choice?”* One participant responded by: *“No*,* I think this freedom of choice would have been too much for me personally. So*,* I think it was a good approach”.* (Gyn_8) Another pointed out that *“Well*,* I thought the surgeon took an incredible amount of time and I’m very grateful to her for that”* (Gyn_15).

Nevertheless, a minority of participants identified areas for improvement. Two participants suggested that earlier diagnostic clarity and more timely communication - particularly regarding pathology results - would have enabled quicker and more confident decision making. One participant maintained “*What you can do to take away people’s fear (.). Speed. Speed might have helped. Speed in pathology would have helped*,* so that a decision could have been made more quickly. (…) In other words*,* the pathology has to fit*,* it has to be right and you have to have confidence in it: It’s all right“* (Gyn_15).

Others indicated a desire for a more holistic approach, including integrative medicine options and continuity of care. Specifically, they expressed a need for a central point of contact who could coordinate across multiple phases of treatment, including surgery and chemotherapy. Fragmented communication and perceived gaps in ownership or responsibility were cited as barriers to feeling fully informed and supported.

For example, one participant said *“[…] But it’s also about the surgery and the follow-up treatment. It’s such a holistic concept and I’ve always had the feeling*,* often the feeling*,* that I only get partial information. Nobody wants to talk to me about the other things. Sure*,* when I’m here for chemo*,* nobody wants to talk about the operation or answer any questions. Or there’s nobody who says okay*,* we’ve now decided this*,* we’ve decided this and this and this will be done”* (Gyn_18).

Overall, the initiation phase revealed a spectrum of needs - from reliance on structured guidance to calls for more comprehensive, integrated care.

### Maintenance phase

In the maintenance phase of treatment, nine participants provided insights into their experiences with ongoing decision making. Much like the initiation phase, participants emphasized the importance of being well informed in order to anticipate and understand the course of treatment. Again, clear explanations and transparent communication emerged as key sources of reassurance and empowerment throughout this period. Many participants expressed a high degree of trust in their care providers and welcomed structured recommendations regarding changes in therapy. They often deferred to medical expertise, citing a sense of security in the guidance offered. For instance, some described accepting treatment decisions without hesitation, relying on the experience and judgment of their clinicians rather than seeking extensive additional information. This dynamic underscores a form of delegated autonomy grounded in trust, rather than active, information-driven deliberation. One participant said: *“We talked about how things could continue afterwards*,* what could be done. […] And I was then immediately told about the possibilities of*,* um*,* antibody therapy*,* that we could incorporate that directly into the chemo. The pills […] that’s. If I’m told: “Take them.” then I take them. I don’t ask a lot of questions. I just don’t want to presume to do that.”* She further continued *“And I’ve always felt well informed”* (Gyn_17). Another participant mentioned, *“[…] I made the decision. I knew what I was getting myself into and what I was about to face. Yes*,* I agreed to it because I understood what was coming”* (Gyn_19).

However, several participants noted limitations in the continuity of communication. In particular, some expressed a desire for repeated or more comprehensive conversations about treatment alternatives, prognosis, and future care options in peace. The absence of follow-up discussions or a central care coordinator sometimes left patients feeling uncertain or under-informed, particularly when navigating end-of-life considerations or exploring additional therapies like integrative medicine. For example, one participant said, *“In between*,* when I think about it*,* it might have been nice if someone had been there to talk to me again in peace*,* right?”* (Gyn_20).

Furthermore, despite efforts to be included in decisions, some participants reported feeling overwhelmed by the complexity of choices, particularly after surgery or when facing multiple treatment options. In these instances, the burden of decision making was experienced as distressing, reinforcing the importance of supportive, clinician-guided dialogue. For example, responses such as *“After the operation*,* the question was: chemo*,* radiation*,* which way?”* (Gyn_15), and *“The problem is*,* that you’re actually so overwhelmed with all these questions and decisions. Of course you want to know and of course you want to be asked*,* but ultimately you can’t make a good decision. You don’t know what to expect. You don’t know what it will feel like after the operation. […] You don’t know what the effects really are. And you can’t make a good decision. You have to rely on the doctors to decide to the best of their knowledge and belief”* (Gyn_15).

Others articulated a deliberate choice to limit their own knowledge out of self-protection, preferring not to be confronted with potentially distressing information: *“Because I can’t cope with life right now. I can’t make a decision. Not a medical one. I can’t.”* (Gyn_16) or elsewhere, *“Maybe it’s also a bit like not wanting to know too much”* (Gyn_18).

Well-conducted informational discussions facilitated treatment acceptance for several participants, likely by improving their understanding of the intervention. In several cases, detailed, empathetic explanations from clinicians helped patients overcome fears - particularly regarding chemotherapy - and fostered a stronger sense of agency. *“During the discussions*,* during the informative talks*,* I was lucky enough to be informed by the doctor. And she was able to tell me so much about this medication and the side effects*,* about the positive aspects of the drug and the chemotherapy*,* that I was suddenly no longer afraid of chemotherapy. […]. Nobody is forcing me to do it and I can do what I want. It’s my life*,* my health. And it was very*,* very important for me to know that I didn’t have to do anything“* (Gyn_20).

Still, a few participants emphasized the need for greater clarity and control, expressing frustration when medical findings lacked transparency or when decisions seemed to prioritize institutional routines over individualized care. For example, one participant mentioned that *“I would have liked to have had the opportunity to question or ask about the findings again or to know what was actually going on” […] “In order to be able to make a decision or to be able to understand a decision well”* (Gyn_15).

In summary, decision making in the maintenance phase was characterized by a complex interplay between clear and transparent information, emotional resilience and trust in clinicians.

### Abstention/ end of treatment

Compared to the more frequently addressed themes of initiating or adapting therapy, decisions related to treatment cessation or abstention were mentioned less often in the interviews. Of the three participants explicitly asked about their thoughts on withholding further treatment, two offered tentative or evasive responses, often redirecting the conversation towards hope and personal coping strategies. This suggests a potential emotional resistance to engaging directly with end-of-life decision making. One participant, for example, hesitated to respond when asked about quality-of-life trade-offs, reflecting uncertainty and a possible preference for selective information as a form of psychological self-protection. Hopeful narratives from other patients or media served as emotional anchors, reinforcing a more future-oriented, resilience-based perspective.

As the recruitment of participants took place in the chemotherapy outpatient clinic of a university medical center, there may be a certain hidden bias here, which will be discussed in the section on limitaions.

One participant addressed the subject of therapy cessation in concrete terms. She emphasized the importance of detailed and honest communication, stating that her decision to continue or forgo further therapy would be contingent on a clear understanding of the risks, benefits, and prognosis. Her account underscored a desire for autonomy that is informed and deliberative, shaped by trust in professional guidance but rooted in personal values and thresholds of acceptability. She said *“Let’s say*,* if you notice that the tumor is starting to grow again or you have to do something else*,* and if it was to be followed by normal chemotherapy*,* I might go along with that a few times. But if you realize that it doesn’t help that much*,* then I think I would decide against it”* (Gyn_20). This response is in contrast with others who preferred to delegate such decisions or shield themselves from difficult prognostic realities.

### Pending

Anticipatory decision making - or decisions that had not yet been made - represented another theme, captured through a variety of concerns ranging from surgical choices and fertility planning to the desire for procedural transparency. Participants’ reflections on upcoming decisions were marked by dual orientation, highlighting the complexity of their decision making process where pragmatic considerations intersected with unresolved emotional responses. On the one hand, they highlighted logistical concerns such as feasibility of surgery or the potential implications for everyday functioning. On the other hand, they expressed affective ambivalence oscillating between hope and worry. For example, one participant expressed fear and uncertainty about the prospect of a mastectomy and the implications of genetic testing: *„I’m just a bit worried about the operation. What it will look like afterwards. And then there’s also the genetic test. And then you’d have to decide whether to have it removed completely or not.”* (Gyn_2). This quotation illustrates how participants sought to reconcile rational evaluations of medical options with emotional uncertainty and fear, revealing an ongoing negotiation between control and loss in the context of genetic risk testing.

Another spoke about the abstract nature of family planning while still undergoing therapy, indicating that the cognitive and emotional load of current treatment limited the ability to project into the future. *„For me*,* that is this: How? It’s too abstract for me right now*,* because I’m still too much in therapy*,* where I also say: “I can’t imagine having children right now.” Because I don’t know at the moment whether I can*,* how certain that is*,* no. For that*,* we need another two or three years where you’re lying in the MRI and they say: “Ms. (patients’ name)*,* we didn’t see anything this time. (…) Yes. Because when I realize*,* when I start thinking about it: Do I want a family? How big should the family be? Then I lose my courage and start to cry. And that doesn’t help me*,* because I can’t decide now.”* (Gyn_16).

The same participant was particularly illustrative requesting to attend the tumor board not to challenge decisions, but to witness the process and assess the care with which decisions were being made. This desire for procedural transparency highlights the importance of relational trust, not just in outcomes, but also in the perceived integrity and attentiveness of the medical decision making process itself. *„And then I said that I wanted to attend the tumor board. I insisted because I wanted to see the people. And I wanted to see the process. Not at all to say: “Sorry*,* I have a different idea. I wanted to be part of the process and see who was making decisions about my life and how they were doing it. How carefully do people do that?”* (Gyn_16).

## Discussion

By revealing gynecologic cancer–specific experiences of shared decision-making, our findings highlight critical opportunities to tailor patient-centered care, offering both new understanding and strong alignment with established oncology research. Our findings suggest a wide spectrum of preferences and needs in the cohort we studied - from a reliance on clinician-led guidance during early stages of the disease, to an increasing desire for more information and autonomy in pending stages towards independent decision making. It can be inferred that the examined patients have different needs and strategies to make decisions not only during different stages of the tumor disease but also on an individual level.

In early decision-making, patient autonomy is often exercised as relationship-based autonomy grounded in trust and collaboration with healthcare providers, rather than as fully independent choice. Supportive, well-coordinated communication is therefore critical to fostering confidence and trust in the treatment trajectory.

Sustained responsive communication and consistent points of contact are essential to supporting patient autonomy and well-being throughout the treatment continuum. Patients often avoided giving direct answers to questions regarding abstinence and instead responded evasively, which we interpreted as a reluctance on the part of participants to engage with this topic. A potential source of bias must be acknowledged: all participants were recruited from a university hospital whose primary mandate is the provision of tumour therapies, whereas patients not undergoing therapy are treated in other settings. Due to a variety of concerns ranging from surgical choices and fertility planning to the desire for procedural transparency, participants reflected on upcoming decisions with a mix of practical anxiety and emotional ambivalence.

Interpreting these findings within the ethics of decision making models in healthcare, particularly in oncology, leads to several critical insights grounded in bioethics, SDM, and patient autonomy. In the following, we briefly list the ethical themes emerging from the analysis and then continue to discuss their implications.

First, our interview data illustrate a nuanced ethical tension between the principles of autonomy and beneficence (doing good). While respect for patient autonomy emphasizes informed choice, control, and active participation in care, beneficence and non-maleficence (not doing harm) often compel clinicians to step in and guide decisions, particularly when patients are emotionally distressed or cognitively overwhelmed. This tension becomes especially salient in the context of SDM, where patient involvement is valued yet must be balanced against the situational demands of the clinical problem at hand - highlighting that SDM is not a uniform procedure but a set of adaptable practices shaped by the specific problems that bring patients and clinicians together [31]. Several participants, including Gyn_8 and Gyn_20, described feeling relieved when medical professionals temporarily assumed responsibility for decision-making. These narratives suggest that an excessive emphasis on autonomy, without sufficient emotional or contextual support, can feel less like empowerment and more like abandonment. Such findings echo O’Neill’s critique of individualistic and procedural autonomy, which warns that treating autonomy as a mere formal requirement fails to account for patients’ situational vulnerabilities and need for relational support [4, 32].

Second, our findings support the contextual relevance of SDM rather than treating it as a unique ideal. While some prior work [6, 33] of SDM imply a single “ideal” way to involve patients, in practice, approaches must be adapted to individuals needs and circumstances. Our interviews revealed tiered or situational models of SDM: some patients (e.g., Gyn_9, Gyn_11, Gyn_15, Gyn_17) actively seek information and involvement whereas others (e.g., Gyn_3, Gyn_20) prefer directive guidance, especially under stress. One way to enhance the situational application of SDM is through Elwyn et al.’s clinical practice model introduced earlier [6, 16]. They present practical guidance for implementing SDM in clinical settings, emphasizing collaboration between patients and healthcare providers. It aims to overcome common barriers by promoting clear communication strategies, fostering patient engagement and supporting clinicians in integrating SDM into routine care. However, the model may oversimplify the complexity of integrating SDM into busy, time-constrained clinical environments (we will revisit this point in the section on limitations).

Another prominent model evident in the data is temporal autonomy, which acknowledges that patients’ readiness to make decisions may evolve over time. Several participants, such as Gyn_16 and Gyn_18, reported feeling emotionally overwhelmed during critical moments, rendering autonomous choice momentarily inaccessible. However, as their emotional resilience grew, so did their engagement in decision making. Autonomy is in this context not static, but temporally situated - requiring ethical consideration of a patient’s psychological and informational readiness at any given time [5]. An important consideration therefore is, that patients should be prepared for SDM. Emerging research has begun to explore strategies to optimize patient readiness for SDM and to identify the most appropriate timing for engaging patients, with the aim of anticipating and mitigating potential patient overwhelm [34]. 

Echoing O’Neill’s critique of procedural autonomy, our findings suggest that the reduction of autonomy to individual consent mechanisms fails to meet the ethical demands of real-world care. In line with her Kantian-inspired view, we propose that autonomy is best exercised when patients are enabled to act on well-informed, principled choices that are embedded in mutual trust. Supporting autonomy, therefore, is not about withdrawing clinician involvement, but about fostering trustworthy relationships and institutional practices that empower patients without abandoning them.

Lastly, and closely related to the previous point, relational autonomy claims that individuals do not make decisions in isolation, but within networks of relationships and sociocultural influence [5]. Our interview data strongly affirms this perspective. Participants frequently referenced support from partners, family members, and friends in navigating complex choices (e.g., Gyn_9, Gyn_15). Furthermore, cultural context appeared to play a significant role in specific situations, as one participant for example observed that women from certain backgrounds tended not to voice their medical preferences.

Against this context, autonomy represents itself as a cornerstone of contemporary healthcare ethics, yet its application in real-world clinical contexts remains complex and contested. By examining the interviewees’ narratives through the lenses of four theoretical models - relational autonomy, temporal autonomy, SDM, and critiques of procedural autonomy - it becomes evident that, first, as Onora O’Neill (2002) has persuasively argued, autonomy must be understood not as isolated self-determination, but as inherently relational [32]. Our data strongly reinforces this view. The decision making narratives reveal that patients do not make choices in isolation; rather, their agency is shaped by a combination of emotional dependencies, family relationships, culturally informed norms and the institutional structures within which care occurs. Participants consistently expressed that autonomy felt meaningful only when supported by clear communication, continuity of care, and trust in the clinical team.

Following scholars of relational autonomy, the data argues against individualistic autonomy which “offers an impoverished or incomplete view of the human condition” [5]. Rather than viewing the self as independent, relational autonomy emphasizes that individuals are situated within networks of relationships-with family, community, and broader society, which influence their identity, capabilities, and decisions. Core elements of relational autonomy include responsibility, mutual care, and interdependence, recognizing that personal development and life choices are continuously formed and informed through these enduring interpersonal connections [5].

However, although relational autonomy provides a robust foundation for interpreting the bulk of the findings, to fully reflect patients’ lived realities, it must be complemented by sensitivity to changing emotional readiness over time and decision-making preferences (e.g. not to decide alone) together with a critical appraisal of structural constraints on autonomy (to address systemic barriers to autonomy).

While relational autonomy rightly accounts for interpersonal influence, the interviews also reveal frustration with institutional and systemic barriers- fragmented care, bureaucratic opacity and lack of access to second opinions. These are not just relational issues but structural injustices, which relational autonomy doesn’t fully theorize, similar findings can also be found in other works [26]. Relational autonomy tends to assume that relationships are supportive, but they can also constrain or manipulate. Additionally, a few participants expressed a preference for clinician-led decisions or reported feeling overwhelmed by an excess of choice (Gyn_3, Gyn_20). For these individuals autonomy was experienced as a relief from responsibility and less as self-determination. This suggests, that sometimes autonomy may be located in the capacity to delegate rather than to decide. This raises questions about whether “relationality” is always a meaningful or desirable focus of autonomy.

Our findings are consistent with qualitative interview studies in breast cancer that delineate marked heterogeneity in patients’ decision-making preferences and highlight the importance of individualized information provision and emotional support as core components of effective SDM [22, 23, 26]. In contrast to this, the qualitative literature in gynecologic oncology remains limited and predominantly centred on specific decision contexts (e.g., fertility preservation) [24]. This thematic narrowness underscores the added value of our study in offering a more comprehensive, patient-centred account of SDM across diverse gynecologic cancer diagnoses within the organisational realities of multidisciplinary decision-making structures.

While the study highlights the centrality of individual clinician-patient encounters in shaping SDM, it pays comparatively less attention to broader systemic or organizational factors that influence these interactions. These include time constraints, reimbursement models, limitations in electronic health records, and institutional culture- all of which can significantly influence the feasibility of implementing SDM in routine practice. As with the Elwyn model, integrating SDM into busy clinical workflows may be more challenging than theoretical frameworks assume, especially in high-pressure oncology settings.

The study offers valuable qualitative insights that contribute to understanding more profound aspects, nevertheless findings need to be validated in a larger sample to determine how generalizable they are.

### Strengths and limitations

The present study offers notable strengths. Methodologicalally, it employs qualitative interviews combined with grounded theory, providing a robust framework for exploring complex patient experiences and decision making processes. The interviews were conducted by a single clinician- researcher, which insured consistency and comparability across the interviews. At the same time the researcher maintained reflexivity to mitigate subjectivity bias, balancing professional familiarity with methodological neutrality and openness – nevertheless this must be recognized as a limitation as discussed below.

The dual role of the lead interviewer as both practitioner and researcher fostered a strong rapport with participants and enabled in-depth discussions of clinically sensitive issues such as side effects, sexuality, fertility, and long-term health priorities. This shared background often enhanced participants’ openness.

This approach increases the credibility and depth of the data collected, particularly through evidential value of the quotes. By including a diverse range of gynecologic tumor entities (ovarian-, cervical-, uterine- and breast cancer), the study attempts to reflect the broad spectrum of clinical practice. Although this heterogeneity may limit tumor-specific conclusions, it is unlikely to affect the overall findings, as the primary objective was to characterize patterns that are broadly relevant across gynecologic malignancies.

Several limitations must be acknowledged. First, the study was conducted at a single-center setting, specifically within a chemotherapy outpatient clinic. This recruitment setting might have introduced selection bias. Patients treated in such a center are likely to be particularly motivated to pursue active treatment, possibly more open to research participation, and may have exhausted standard therapeutic pathways elsewhere. These characteristics could influence their engagement with decision making and may limit the generalizability of findings to other patient populations, including those who are more hesitant about continued treatment or more inclined to consider palliative care or treatment abstention.

Second, given that all interviews were conducted by a single lead interviewer, the potential influence of individual positionality and interpretive bias represents an inherent limitation. To address this, reflexivity was actively maintained through ongoing reflexive journaling, systematic documentation of analytic decisions, and regular supervisory debriefings within the research team. However, since only one interviewer was involved, there was no opportunity to compare and balance different perspectives across interviewers.

Another notable limitation of the present study is that participants were interviewed only once. Consequently, certain aspects of their accounts of decision-making were retrospective and may be influenced by memory biases, while other aspects were prospective, reflecting anticipated experiences rather than events they had encountered. To address this, future research should employ longitudinal designs with interviews of participants at multiple time points, enabling a more nuanced understanding of decision-making as it unfolds over time.

Men with breast cancer and transgender men were not explicitly excluded from this study. However, the final cohort consisted exclusively of cisgender women due to the characteristics of the patient population available at the time of recruitment. Consequently, this is another limitation of our study as the generalizability of our findings to men with breast cancer or transgender men is limited and should be examined in future research.

Another potential limitation is that our sample may overrepresent participants with higher health literacy or familiarity with research, who may be more comfortable discussing clinical topics (sociocultural and educational bias). This could bias findings toward the perspectives of more articulate or research-engaged patients, underrepresenting those with lower literacy, limited access or less comfort in clinical dialogue. Consequently, this potentially limits the generalizability of our results.

Another notable limitation is the absence of an in-depth discussion about clinicians themselves. The study does not address whether all clinicians possess or can easily develop the communication skills required for high-quality SDM. Without adequate training, support, and institutional incentives, even well intentioned clinicians may struggle to engage patients effectively. Future work should examine not only patient readiness for SDM but also the structural and educational needs of clinicians tasked with facilitating it.

In sum, this study provides insights into the experiences of female patients with shared decision making in a single oncological centre in Europe, particularly in the context of advanced disease and complex treatment choices. However, the findings should be interpreted with caution in light of the methodological and contextual constraints and the specific clinical context mentioned above. Further research in more diverse and multi-center settings, along with parallel investigations into clinician capacities and systemic barriers, will be critical to advancing the integration of SDM in oncology care.

## Conclusion

This study provides nuanced insight into how women with gynecologic cancers experience SDM across various stages of their treatment. By applying a grounded theory approach to patient narratives, we uncovered a dynamic spectrum of preferences and needs - from a reliance on clinician-led guidance during initial diagnosis, to increasing desire for information and autonomy during treatment maintenance, and finally, to more emotionally complex and selective engagement with end-of-treatment decisions. These findings reflect patients currently receiving treatment at a university hospital. However, these patterns may differ among individuals who have chosen to modify or discontinue their care elsewhere.

Our findings support an understanding of autonomy as both relational and temporal: patients’ decision making capacities and desires for involvement depend of the stage of disease and are shaped by emotional resilience, interpersonal relationships, and trust in the clinical team. Rather than questioning SDM as a normative ideal, this study highlights the need for an adaptable model capable of responding to diverse patient needs and situational contexts. Trust, continuity of care, and clear, empathetic communication emerge as critical enablers of patient empowerment, particularly in high-stakes, emotionally charged clinical contexts like oncology.

At the same time, our analysis reveals systemic gaps that can undermine the effective practice of SDM. Fragmented communication, time constraints, and the absence of centralized care coordination continue to limit patients’ ability to participate meaningfully in decisions. While the study emphasizes individual interactions, future work must also address broader organizational and structural barriers to implementing SDM in a consistent, patient-centered manner.

## Supplementary Information

Supplementary Material 1.

Supplementary Material 2.

## Data Availability

The datasets generated and analysed for the study are not publicly available due to patient-related data protection regulations. However, they are available upon request in anonymized format.

## Abbreviations

GDPR/DSGVO: European general data protection regulation
Gyn_X: Number of interview perfomed in gynecology
HTA: Health Technology Assessment
PtDAs: Patient decision aids
SDM: Shared decision making
WHO: World Health Organization
