# Supplementary material for: From consultation to choice: gynecologic cancer patients’ perspectives on shared decision making in clinical practice – findings from a cross-sectional observational qualitative interview study
Source: BMC Med Ethics. 2026 Feb 24;27:46. doi: 10.1186/s12910-026-01419-1 (PMC12954969; doi:10.1186/s12910-026-01419-1)
Supplement: Supplementary file 2 — Supplementary Material 2. [file 12910_2026_1419_MOESM2_ESM.pdf]

Supplementary file 2: Consolidated criteria for reporting qualitative studies (COREQ): 32-item checklist

No	Item	Guide questions/description	
**Domain 1: Research team and reflexivity**			
Personal Characteristics			
1.	Interviewer/facilitator	Which author/s conducted the interview or focus group?	ST
2.	Credentials	What were the researcher's credentials? *E.g. PhD, MD*	MD
3.	Occupation	What was their occupation at the time of the study?	specialist physician transitioning to senior physician
4.	Gender	Was the researcher male or female?	Female

No	Item	Guide questions/description	
5.	Experience and training	What experience or training did the researcher have?	Specialist at a university hospital with additional qualification in drug-based tumour therapy, specialising in reproductive medicine.
Relationship with participants			
6.	Relationship established	Was a relationship established prior to study commencement?	Most of the patients and the interviewer did not know each other beforehand. Those who were acquainted were not known from the oncological context, but rather from fertility counselling.
7.	Participant knowledge of the interviewer	What did the participants know about the researcher? e.g. *personal goals, reasons for doing the research*	Participants received an information sheet in advance about the study, its objectives and the procedure. Once they agreed to participate, the study objectives were explained to them again. The participants knew that the interviewer was a doctor at the treating clinic and also a researcher in the interdisciplinary project on gynaecology, internal medicine and ethics.
8.	Interviewer characteristics	What characteristics were reported about the interviewer/facilitator? e.g. *Bias, assumptions, reasons*	Professional position: may lead to authority relation (power imbalance)<br>Interviewer may assume shared knowledge or experiences: might reduce level of probing and clarification<br>Medical treaining and professional perspective of gynegologist maight lead to biomedical framing of questions and potentially overlook social, cultural, emotional dimensions of patients narratives.

No	Item	Guide questions/description	
		and interests in the research topic	Dual role might also support trust, confidentiality, legitimacy in sharing sensitive information<br>Gender concordance interviewer/ participants might foster openness and empathy, enhancing the depth of responses<br>Data may be shaped by professional bias and assumptions
**Domain 2: study design**			
Theoretical framework			
9.	Methodological orientation and Theory	What methodological orientation was stated to underpin the study? *e.g. grounded theory, discourse analysis, ethnography, phenomenology, content analysis*	Grounded theory
Participant selection			
10.	Sampling	How were participants selected? *e.g. purposive,*	Purposive

No	Item	Guide questions/description	
		convenience, consecutive, snowball	
11.	Method of approach	How were participants approached? e.g. *face-to-face, telephone, mail, email*	Face-to-face, telephone
12.	Sample size	How many participants were in the study?	20
13.	Non-participation	How many people refused to participate or dropped out? Reasons?	3 refused (no time/ too many appointments/ not interested), 0 dropped out (cross-sectional design)
Setting			
14.	Setting of data collection	Where was the data collected? e.g. *home, clinic, workplace*	Clinic
15.	Presence of non-participants	Was anyone else present besides the	No.

No	Item	Guide questions/description	
		participants and researchers?	
16.	Description of sample	What are the important characteristics of the sample? *e.g. demographic data, date*	age 31 to 72 years (median 50,5 years, mean age 49.4 years), married (65%), divorced 10%, single 25%. 60% have children, average of 1.3 children per participant (range: 1 to 4). 20% high school diploma (13 years of school), 40% intermediate school-leaving certificate (10 years of school), 30% university levels, 5% primary school or technical college qualification (4 to 9 years of school).
Data collection			
17.	Interview guide	Were questions, prompts, guides provided by the authors? Was it pilot tested?	Semi-structured interview guide. The interviewers had two practice sessions with simulated patients beforehand.
18.	Repeat interviews	Were repeat interviews carried out? If yes, how many?	No repeat interviews were performed.
19.	Audio/visual recording	Did the research use audio or visual	Audio recording was used throughout the interviews,

No	Item	Guide questions/description	
		recording to collect the data?	
20.	Field notes	Were field notes made during and/or after the interview or focus group?	Yes, after / during interviews and in focus groups.
21.	Duration	What was the duration of the interviews or focus group?	Duration of the interviews varied between 15 and 52 minutes with a mean duration of 32 minutes.
22.	Data saturation	Was data saturation discussed?	Yes, it was discussed in regular meetings of the study team.
23.	Transcripts returned	Were transcripts returned to participants for comment and/or correction?	No, transcripts were not returned but they were pseudonymized.
**Domain 3: analysis and findings**			
Data analysis			

No	Item	Guide questions/description	
24.	Number of data coders	How many data coders coded the data?	Two.
25.	Description of the coding tree	Did authors provide a description of the coding tree?	Yes. See results section line second paragraph.
26.	Derivation of themes	Were themes identified in advance or derived from the data?	The question addressed in this paper was derived from the results of the interviews.
27.	Software	What software, if applicable, was used to manage the data?	MAXQDA
28.	Participant checking	Did participants provide feedback on the findings?	The results of the study will be published publicly so that every participant can access them. Participants were given the contact details of the interviewer and the study director for specific queries. No further contact has been recorded to date.
Reporting			
29.	Quotations presented	Were participant quotations presented	Yes.

No	Item	Guide questions/description	
		to illustrate the themes / findings? Was each quotation identified? e.g. *participant number*	
30.	Data and findings consistent	Was there consistency between the data presented and the findings?	This response is the responsibility of the reviewers/editors.
31.	Clarity of major themes	Were major themes clearly presented in the findings?	This response is the responsibility of the reviewers/editors.
32.	Clarity of minor themes	Is there a description of diverse cases or discussion of minor themes?	This response is the responsibility of the reviewers/editors.
